# A systematic review on personalization of treatment components in IBIs for mental disorders

**DOI:** 10.1016/j.invent.2025.100840

**Published:** 2025-06-14

**Authors:** Carmen Schaeuffele, Pavle Zagorscak, Vladlena Langerwisch, Johanna Wilke, Yana Medvedeva, Christine Knaevelsrud

**Affiliations:** a Freie Universität Berlin, Department of Education and Psychology, Clinical Psychological Intervention, Germany; b German Center for Mental Health (DZPG), partner site Berlin-Potsdam, Germany

**Keywords:** Personalization, Tailoring, Internet-based interventions, Adherence, Mental health

## Abstract

**Background:**

Internet-based interventions (IBIs) offer the potential for personalization through various mechanisms and components.

**Objective:**

This systematic review aimed to synthesize evidence on the personalization of treatment components within IBIs targeting diverse mental health conditions. Specifically, we focused on studies that directly compared personalized components to standardized ones to isolate the impact of personalization on mental health outcomes and treatment adherence.

**Results:**

Thirteen studies were identified that compared personalized to non-personalized components, with the personalization of IBI content and personalized guidance investigated the most. Apart from one study that personalized more than one IBI component, studies did not find a significant positive effect of personalization on mental health outcomes. Two studies reported better adherence for human feedback personalized to user input than for the automated non-personalized guidance.

**Discussion:**

The results reveal a gap between the theoretical potential of personalization in IBIs and the current evidence supporting its impact on outcomes and adherence. The diversity in personalization strategies across studies complicates the ability to draw definitive conclusions. To address this, more detailed descriptions of how personalization is both implemented and communicated to patients are recommended.

## Highlights

• Componts of Internet-based interventions (IBIs) can be personalized independently from each other
• This systematic review revealed a limited number of studies that compared personalized components of IBIs to standard components
• Findings highlight a gap between personalization's anticipated benefits and its current empirical support

## 1 Introduction

Mental disorders are highly prevalent (Jacobi et al., 2014). Health care systems face a significant treatment gap to help meet the demand for adequate care: Limited resources in psychotherapeutic care with long waiting times (Van Dijk et al., 2023) as well as personal barriers are obstacles to access mental health care (Thornicroft et al., 2017). Internet-based interventions (IBI) have the potential to help meet the demand, as they facilitate dissemination of effective treatments and address personal barriers, including fear of stigmatization, location, or time (Seiferth et al., 2023). Various terms are used in the literature to describe these interventions, such as e-mental health, guided self-help or web-based interventions (Smoktunowicz et al., 2020). In this manuscript, we use the term IBI to refer specifically to structured, internet-delivered psychological interventions including web- and app-based interventions with or without guidance but excluding formats such as videoconferencing or teletherapy. Most mental health care treatments, including IBIs, follow a “one size fits all” approach. Personalizing interventions (“my size fits me”) may be of benefit to match individual needs, resulting in better effects and adherence (Schaeuffele et al., 2021). Personalization of psychological treatments has been defined as approaches “to select, adapt, or adjust treatment to an individual with the goal of improving outcomes” (Deisenhofer et al., 2024, p. 2). A recent systematic review on definitions of personalization synthesized that personalization aims “to optimize treatment outcome for the individual patient by tailoring treatment to unique or specific needs, preferences or other characteristics of an individual patient and includes a systematic adaptation of treatment or a differentiation between treatment strategies” (Harnas et al., 2024, p. 10). In IBIs, personalization has been characterized as “purposefully designed variations between individuals in an intervention's therapeutic elements or structure” (Hornstein et al., 2023, p. 6)**.** Personalization is often used synonymously with tailoring or individualization and refers to adaptations on an individual - not group - level (Bennett and Shafran, 2023).

### 1.1 Which components are personalized how and when?

With the aim to systematize reporting of personalization of IBI components, Hornstein et al. (2023) recently proposed a framework for IBIs for depression. Their suggested framework differentiates between the levels and mechanisms through which the personalization occurs: In IBIs several components (dimensions, levels, or aspects) of treatment can be distinguished, the (1) content (“variability in the delivered intervention material” e.g., personalized selection of modules), (2) order (“same content but in different order” e.g., personalized order of modules), (3) guidance (“extent of therapeutic support offered” e.g., personalized frequency of guidance), and (4) communication (“channel, timing and content of actively offered information outside of the intervention's content” e.g., personalized reminders). This personalization can be achieved through (1) user choice (“direct choice of the participant”), (2) provider choice (“either the individual providing guidance, or the clinician involved”), (3) rule-based (“if-then-decision rules”), and (4) machine learning models (“decisions with ‘learned’ decision criteria”).

**Fig. 1:**
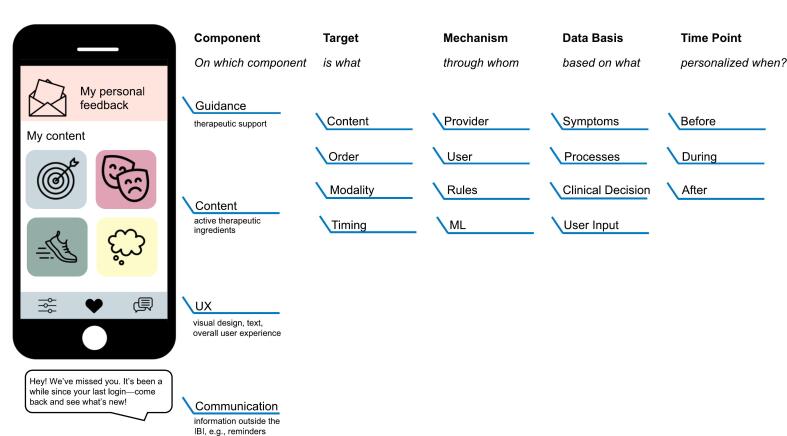
Overview of personalization possibilities of treatment components in IBIs Note. IBIs can be broken down into different components. The components and mechanism are based on Hornstein et al. (2023)'s framework. The component “UX” was added because the other components do not capture variations related to the overall user experience. Different targets of the components may be personalized, e.g., is the guidance personalized in regard to the content (individualized texts), timing (weekly or biweekly,) or modality (text or phone), although not all targets may be relevant for all components. Harnas et al. (2024) have suggested considering the basis of the mechanism. Cohen et al. (2021)'s 3D model of personalization proposed to consider the timepoint of personalization.

While this framework provides an important starting point, more granularity may be needed to fully capture the range of personalization opportunities in IBIs: Harnas et al. (2024) proposes that studies should also report the basis of the personalization mechanism, e.g., whether clinicians personalize treatment modules based on clinical diagnoses or patient preferences. Other models of personalization also consider the timepoint of personalization, i.e., is personalization decided once or dynamically during treatment (Cohen et al., 2021). Considering the variety of these possibilities – level and mechanism (Hornstein et al., 2023), the basis of the mechanism (Harnas et al., 2024), the timepoint (Cohen et al., 2021) as well as the target of the level and complexity – may allow for a more in-depth understanding of personalization in IBIs. Fig. 1 illustrates how the proposed dimensions in the literature could be integrated with each other. We added a component to Hornstein et al.'s (2023) framework that captures how the intervention is tailored in terms of its UX, including visual design, text, and overall user experience. This component can include elements such as the customization of colors, layout, avatars, and other aesthetic features, as well as the way content is presented and interacted with, e.g., the addition of personalized case examples. While the latter could be conceptualized as content, we suggest to capture it in this component to differentiate from personalization of active therapeutic ingredients. While customization may not be considered personalization (Hornstein et al., 2023), Six et al. (2022) demonstrate that a stronger identification and connection with one's avatar is indeed associated with greater symptom reduction. This finding suggests that customization should not be excluded from the spectrum of potential personalization strategies. This proposed framework also combines the levels content and order (Hornstein et al., 2023) to the component “content” as we propose to distinguish between the different targets of personalization per component. For instance, personalization of the guidance component could have different targets, including the actual content of the guidance (e.g. individualized vs. standard feedback), the timing (e.g., fixed weekly vs. flexibly), or modality (text- or video-based). Note that not all targets may be relevant for all components. Additionally, it may be worthwhile to consider the extent and complexity of the implemented personalization. Personalization can range from relatively simple adjustments, such as the use of a personalized avatar, to highly complex interventions that intricately integrate elements of an individual's unique circumstances into every IBI component.

### 1.2 Evidence on the effects of personalization

Several reviews or meta-analyses have investigated dimensions of IBIs and their impact on effects and adherence. Guidance is the best researched dimension of IBIs. Several recent systematic reviews compared individualized (human) to standardized (automated) support (Koelen et al., 2022; Shim et al., 2017; Werntz et al., 2023). Individualized support seems to be associated with small incremental effects for outcomes and adherence in comparison to standardized support (Koelen et al., 2022). However, in an individual participant data meta-analysis on IBIs for depression, Karyotaki et al. (2021) found that guidance is not generally associated with better outcomes but that patients with more severe symptoms at baseline may benefit from guidance. This highlights the potential for personalization, for example in regard to the intensity of support in the context of IBIs. On the dimension of content or order of modules, Păsărelu et al. (2017) found that tailored approaches showed moderate effects on anxiety and depression but they did not investigate whether tailored approaches are more beneficial than standard treatment approaches. Nye et al. (2023) compared personalized to standard psychological therapies. While there was no dedicated focus on IBIs in their systematic review, they identified two older studies that compared a personalized module selection and sequencing within an IBIs with standard IBI versions (Berger et al., 2014; Johansson et al., 2012). Across all treatment formats, they included nine studies in the meta-analytic calculations and found that personalized approaches can increase the efficacy of standard approaches with small effects (*d* = 0.22). Given that the search strategy was not focused on digital approaches, additional studies on personalized IBIs may have been overlooked. Tailoring is also recommended as a potential strategy to increase adherence (Karekla et al., 2019). Perceiving an IBI as a good fit may foster engagement (Borghouts et al., 2021). However, the impact of personalization of treatment components on adherence has not been systematically investigated (see Beatty and Binnion, 2016 for a systematic review of predictors of adherence in IBIs).

### 1.3 Aim for this systematic review

For the purposes of this review, personalization is defined as the individual-level purposeful adaptation of one or more IBI components through various mechanisms, as IBIs components can be personalized independently from each other. While Hornstein et al. (2023)'s review highlighted that personalization has been widely applied in IBIs, the current state of research is marked by several significant limitations. Existing studies report inconsistent findings regarding the effects of personalization, with many focusing narrowly on specific dimensions. While the IBI components guidance and content have been explored to some extent, the comparative efficacy of personalized versus standardized approaches remains unclear. Moreover, the influence of personalization on adherence - a critical factor for the success of IBIs - has not been systematically examined, leaving a key gap in understanding if and how personalized approaches can foster engagement. These shortcomings underscore the need for a more comprehensive and nuanced evaluation of personalization in IBIs. Our systematic review aims to address these gaps by synthesizing the implementation and evidence on personalization across diverse mental health conditions. By utilizing the above outlined definition and components —guidance, content, communication, and UX —, we seek to provide a nuanced and actionable understanding of personalization in IBIs, moving beyond the limitations of previous reviews to inform both research and practice. To isolate the potential benefit of personalization on these different levels, this systematic review will focus on studies that compared personalized to standardized components and synthesize the effect of personalization on mental health outcomes as well as adherence.

## 2 Method

We followed the Cochrane Handbook for systematic reviews (Higgins et al., 2024) and the updated Preferred Reporting Items for Systematic Reviews and Meta-Analyses (PRISMA; Page et al., 2021) to report this systematic review. The protocol was pre-registered on Prospero (registration number: CRD42024501774).

### 2.1 Search strategy

Table 1 displays the inclusion and exclusion criteria following the PICOS framework. We conducted a systematic literature search on the libraries PubMed and MEDLINE, PsycINFO, medRxiv (incl. bioRxiv), and OSF Preprints up to 13/03/2025. The exact search strings for the respective libraries are provided in the appendix and comprise a combination of “personalization” and “Internet-based Intervention”, “Randomized Controlled Trial” and “mental disorder” using the AND boolean operator. For each concept, we included multiple, synonymously used terms with the OR boolean operator. We applied relevant Medical Subject Headings (MeSH) terms to index articles in PubMed and MEDLINE as well as terms commonly used in the relevant literature (e.g., “tailored”). We also hand-searched the reference lists of relevant studies and included additional studies if they met our inclusion criteria (Table 1).

**Table 1:** Inclusion and exclusion criteria according to the PICOS framework.

	Inclusion criteria	Exclusion criteria
Population	Adults with elevated symptoms of a mental disorder (established with self-report or clinician-rated measures based on cut-offs) and/or a mental disorder (self-reported or diagnosed by a clinician or according to a diagnostic interview).	Studies involving children or adolescents Studies involving populations with a primary somatic disorder
Interventions	Internet-based interventions delivered through digital platforms, websites, or apps With personalized components (e.g., tailored content, personalized feedback) Targeting mental health outcomes	Non-internet-based interventions (e.g., face-to-face therapy, phone-based interventions, blended treatments) Interventions without personalized components
Comparator	Standard treatment or treatment component	Interventions without standard equivalent of the personalized treatment or treatment component
Outcomes	Adherence outcomes (e.g., completion rates, frequency of logins, engagement metrics) or Self-report or clinician-rating of mental health outcomes (e.g., changes in symptoms of depression or anxiety)	studies not reporting adherence or specific mental health outcomes
Study Design	Randomized controlled trials	Meta-analyses, systematic reviews Studies in languages other than English or German

### 2.2 Study selection and data extraction

Two reviewers independently screened the search results in duplicate, first based on title and abstract. Full-text articles were then also assessed independently by two reviewers to determine eligibility. The screening process was facilitated by the online tool Rayyan (https://www.rayyan.ai/) which supports blinded, independent review and highlights conflicts of independent ratings. In cases of disagreement, conflicts were discussed and resolved within the research team, involving CS and PZ. Interrater-agreement was reached for 95.6 % of the reviewed titles/abstracts. Data extraction was conducted using a standardized extraction sheet. Initial data extraction was performed by one member of the research team and subsequently checked for accuracy and completeness by CS. Extracted information included study design, participant characteristics, intervention and comparator details, personalization features, outcome measures, and main findings. The extraction sheet is provided on the Open Science Framework (OSF) at https://osf.io/u74hw. Any uncertainties or discrepancies were discussed within the research team and resolved by consensus.

### 2.3 Study quality assessment

For RCTs, we evaluated the risk of bias of the studies by using the revised Cochrane risk-of-bias tool (RoB 2.0) (Higgins et al., 2019). We rate the risk as “low”, “some concerns”, or “high” in the following five domains: (a) bias of the randomization process; (b) bias of deviations from intended interventions; (c) bias of missing outcome data; (d) bias in measurement of the outcome; and (e) bias in selection of the reported results. We visualized the RoB 2.0 with the R package robvis (McGuinness and Higgins, 2020). We documented the review process with a PRISMA Flowchart.

## 3 Results

### 3.1 Study selection

**Fig. 2:**
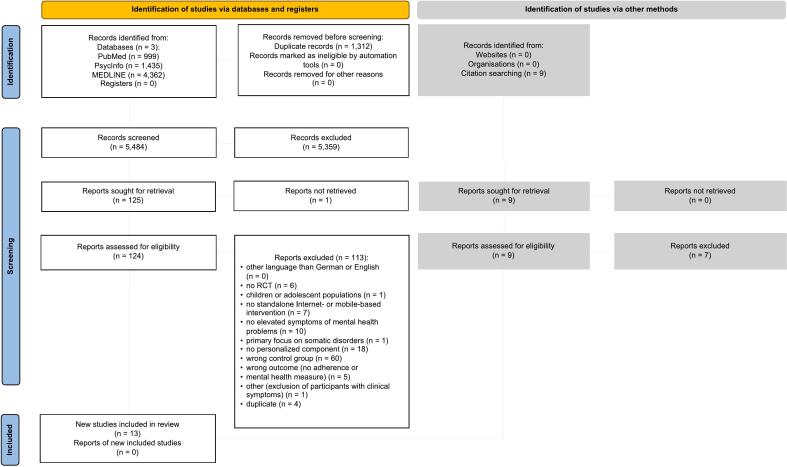
PRISMA flow.

We identified 6796 articles (see flow chart in Fig. 2). After removing duplicates, we screened 5484 abstracts of which 124 studies were full-text screened. Reasons for exclusion are provided in table S2 in the appendix. Finally, we identified thirteen studies comparing personalized components in IBIs to non-personalized components.

### 3.2 Characteristics of included studies

We included *k* = 13 RCTs which investigated at least one personalized component on any level. Table 2 provides an overview of the study characteristics. Our review comprised *n* = 1765 participants who received a personalized IBI component and *n* = 1664 who received a non-personalized component. Studies were conducted in Western countries. All studied interventions were based on cognitive-behavioral therapy (*k* = 13). *K* = 5 IBIs followed a transdiagnostic approach. The number of modules varied between 4 and 10 with a median of 7 modules. Personalization was most commonly implemented on the content (*k* = 6) and guidance component (*k* = 6). *K* = 2 personalized the UX. No study personalized communication (*k* = 0). *K* = 1 studies personalized more than one component.

**Table 2:** Study characteristics.

										Personalization of				
Author	Country	N Personalized	N Standard	%Female Personalized	%Female Standard	Targeted Problem	Number of Modules	Therapeutic Focus	Therapeutic Orientation	Content	Order	Guidance	Communicati	UX
Batterham et al. (2017)	Australia	66.0	62.0	88.0	81.0	depression, anxiety, suicidal ideation, substance use	10	transdiagnostic	CBT	yes	no	no	no	no
Berger et al. (2011)	Switzerland	27.0	27.0	55.6	48.1	social anxiety	5	disorder-specific	CBT	no	no	yes	no	no
Berger et al. (2014)	Switzerland, Germany, Austria	44.0	44.0	59.1	54.5	anxiety disorders (social anxiety, panic disorder, generalized anxiety disorder)	8	disorder-specific	CBT	yes	no	no	no	no
Johansson et al. (2012)	Sweden	39.0	40.0	74.4	70.0	depression with comorbid symptoms	10 weeks (8 sessions in standard treatment; up to 10 sessions in personalized treatment)	not specified	CBT	yes	yes	no	no	no
Kelders et al. (2015)	Netherlands	151.0	88.0	59.1	77.5	depression	9	transdiagnostic	CBT	no	no	no	no	yes
Kleiboer et al. (2015)	Netherlands	108.0	106.0	64.0	68.0	anxiety and/or depression	5	transdiagnostic	CBT	no	no	yes	no	no
Koelen et al. (2024)	Netherlands	269	265	71.5	71.5	anxiety and/or depression	7	transdiagnostic	CBT	no	no	yes	no	no
Linardon et al. (2023)	Australia	77.0	78.0	94.8	94.9	binge eating	4	disorder-specific	CBT	yes	no	no	no	no
Niles et al. (2020)	USA	336.0	323.0	NA, general %female: 80.0	NA, general %female: 80.0	PTSD	12 sessions of which each included 90 trials	not specified	CBT	yes	no	no	no	no
Nissling et al. (2021)	Sweden	28.0	28.0	63.0	78.6	anxiety disorders	8	transdiagnostic	CBT	yes	yes	yes	no	no
Six et al. (2022)	USA	45.0	49.0	72.0	73.0	depressive symptoms	7	disorder-specific	CBT	no	no	no	no	yes
Sundström et al. (2016)	Sweden	20.0	20.0	60.0	75.0	alcohol use	8	disorder-specific	CBT	no	no	yes	no	no
Zagorscak et al. (2018)	Germany	555.0	534.0	64.9	66.3	depression	7	disorder-specific	CBT	no	no	yes	no	no

### 3.3 Risk of bias rating

**Fig. 3:**
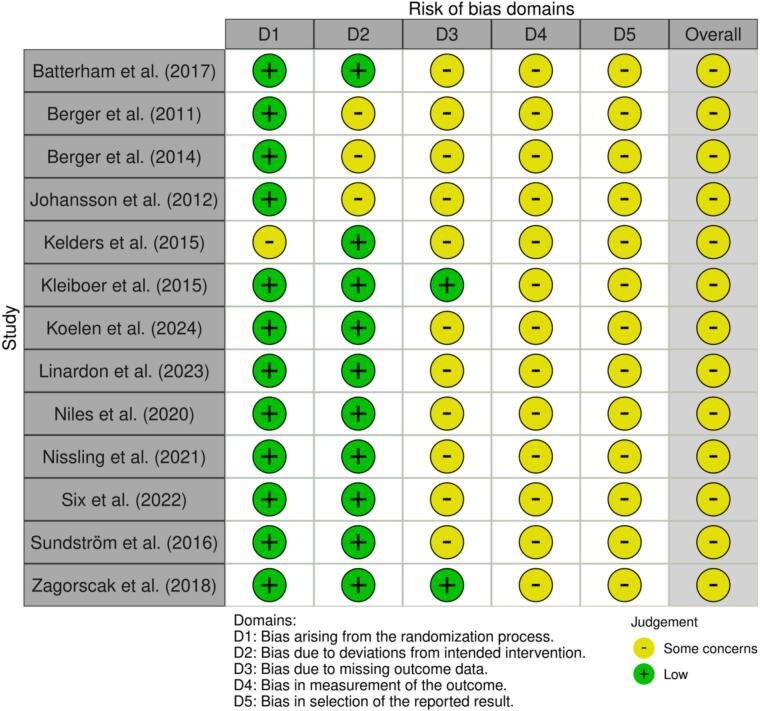
Risk of bias assessment. Note. Traffic-light plot of the domain-level judgements. Risk of bias was assessed across five domains for each study included in the meta-analysis using the revised Cochrane risk-of-bias tool (RoB 2.0). The combination of assessments in the five domains results in an overall risk of bias rating.

All (*k* = 13) studies were rated as having at least some concerns regarding their overall risk of bias. A traffic plot of the RoB 2.0 assessment is depicted in Fig. 3. All studies except one (*k* = 12, 92.3 %) were rated as having a low risk of bias in relation to the randomization process. One study (k = 1, 7.7 %) reported problems with randomization that resulted in unequal group sizes (Kelders et al., 2015), along with some baseline differences between groups. For deviations from intended interventions, *k* = 10 (76.9 %) studies were rated as having a low risk of bias in this domain. Participants and/or personnel were likely aware of their assigned intervention in all studies (k = 13), which is common in psychological intervention research. Three studies (k = 3, 23.1 %) used outdated intention-to-treat approaches, contributing to a rating of some concerns in this domain. Regarding bias due to missing outcome data, k = 2 (15.4 %) studies were rated as having low risk of bias, as they conducted additional analyses to assess the potential impact of missing data. The remaining k = 11 (84.6 %) were rated as having some concerns in this domain. The domain of measurement of the outcome was rated as having some concerns in all studies (k = 13), as all outcomes were based on self-report measures. Finally, for selection of the reported results, all studies (*k* = 13) were rated as having some concerns. Only one study (*k* = 1, 7.7 %) specified that an analysis plan was developed in advance (Niles et al., 2020), though it was never published.

### 3.4 Personalization of content

Content level personalization was implemented in *k* = 6 studies. Table 3 provides a summary table of the personalization characteristics and overview of findings. Personalization of content entailed a range of personalization. Most commonly it entailed a personalized selection of treatment modules through different mechanisms and different bases: Either patient-driven module selection and order in a mixed anxiety disorder sample (Nissling et al., 2021), rule-based module selection based on symptom profiles in a transdiagnostic sample with depression, anxiety, suicidal ideation, and substance use (Batterham et al., 2017) or mixed anxiety disorders (Berger et al., 2014), or clinician-selected modules in personalized order based on self-reports and SCID diagnoses in a depressed sample with comorbid complaints (Johansson et al., 2012). However, personalization of content also comprised patients choosing between two different IBIs in patients with eating disorders (Linardon et al., 2023) or a personalized selection of words in an attention bias modification training (Niles et al., 2020). Except for Nissling et al. (2021), none of the identified studies reported a superiority of personalized vs. standard content. In Nissling et al. (2021)'s study, participants in the personalized, patient-driven condition had a significantly larger reduction in anxiety compared to standard treatment. However, patients not only chose the IBI treatment, the modules and their order but also the frequency and modality of guidance. Johansson et al. (2012) reported that a subgroup with higher initial depression showed that tailored treatment was more effective than the standardized treatment, both in terms of reduction of depressive symptoms and on recovery rates. No study reported any indication that personalization of content significantly improved adherence.

**Table 3:** Summary table of personalization of content.

Study	Target Population	Personalization Mechanism	Basis	Comparison Condition	Effect on Outcomes	Narrative Summary on Outcomes	Adherence Definition	Effect on Adherence	Narrative Summary on Adherence
Nissling et al. (2021)	anxiety disorders	Patient-selected IBI, modules, order, guidance modality & frequency	Patient preference	standard IBI program for anxiety disorders with standard weekly written guidance	Significant difference in anxiety	Significant difference in anxiety at post-treatment but not on other symptoms. At 3-month follow up, personalized group showed larger reduction of symptoms in the observed values, with between-group effects of d = 0.33 for anxiety symptoms, d = 0.47 for depression and d = 0.56 for general disability.	% of modules ompleted	No significant effect	% of modules completed appeared comparable between groups: 12 participants (48 %) in the personalized group completed 75 % to 100 % of modules compared with 14 (50 %) in the standard group.
Batterham et al. (2017)	depression, anxiety, suicidal ideation, substance use	Rule-based module selection	Symptom profile	Standard modules	No significant effect	No difference in symptom reduction between conditions both in primary outcome (composite score) nor specific symptom domains.	Number of modules completed	No significant effect	Module completion was descriptively slightly higher in the personalized group (M = 4.0, SD = 3.7) than the standard group (M = 3.6, SD =3.4).
Berger et al. (2014)	Mixed anxiety disorders	Rule-based module selection	Symptom profile	Standardized intervention	No significant effect	Treatments did not differ significantly on any symptom measures and the effect size between both groups were neglibly small (Cohen's d between 0.00 and 0.02)	Completed treatment; time spent	No significant effect	Descriptively slightly higher completion rate in the personalized group (personalized vs. standard: 75 % vs. 70.9 %,); participants also spent more time on the platform (personalized vs. standard: M = 22h28m vs. 18h13m), potentially due to access to more content within sessions.
Johansson et al. (2012)	Depression with comorbidity	Clinician-selected modules and order	SCID & self-report	Standard treatment	Positive effects for subgroup with high depression	Tailored treatment was not more effective than standard treatment in primary or secondary outcomes. Subgroup analysis of a sample with higher initial depression showed that personalized treatment was more effective than the standard treatment, both in terms of reduction of depressive symptoms (between group effect sizes of d = 0.69–0.82 for depression measures) and on recovery rates (50 % recovery for personalized vs. 17.6 % for standard group)	Number of finished modules of prescribed modules	No significant effect	No significant difference in module completion rates between personalized (77.2 %) and standard treatment (80.7 %)
Linardon et al. (2023)	Eating disorders	Choice between two IBIs	Patient preference	Random assignment	No significant effect	Between-group comparisons revealed small to medium non-significant differences between choice and no choice	Number of self-monitoring diary entries; number of modules/sections completed; completion of at least 50 % of program content; frequency of accessing program; dropout	No significant effect	No significant difference between choice and no-choice group on the number of self-monitoring diary entries, number of modules/sections completed and completion of at least 50 % of program content; participants in choice condition accessed program on more days than in no-choice condition (due to type of program instead of group allocation)
Niles et al. (2020)	PTSD	Personalized word stimuli in ABM	Self-reported concerns via ML	Non-personalized words	No significant effect	No significant Time × Condition interaction; only the personalized ABM group showed a within-group reduction in anxiety from baseline to posttraining	Training sessions completed	No significant effect	No significant differences were found in initial engagement among the treatment groups

### 3.5 Personalization of guidance

Personalization on the dimension of guidance was implemented in *k* = 6 studies. Personalization of guidance entailed a personalization on the following targets: timing, modality, as well as the therapeutic content of guidance. Table 4 provides a summary table of the personalization characteristics and overview of findings. In two studies (Berger et al., 2011; Kleiboer et al., 2015) patients could request contact with a coach/therapist after completion of every lesson, while participants in the control group were contacted once per week after lesson completion, regardless of their preferred guidance frequency. In Nissling et al. (2021)'s study, patients had the flexibility to personalize their experience by selecting not only the content and sequence of their sessions but also the frequency of support (up to once per week) and the preferred modality, either written or via telephone. Sundström et al. (2016) exclusively investigated the modality of guidance in patients with alcohol use disorder: Patients had a choice between guidance via asynchronous text messages or synchronous text-based chat. Zagorscak et al. (2018) and Koelen et al. (2024) adapted the content of the feedback to the individual person and compared this personalized feedback with standard automated feedback. No study, apart from Nissling et al. (2021) (see above), reported any positive impact of personalization of guidance (frequency, modality nor personalized feedback) on outcomes. Zagorscak et al. (2018) and Koelen et al. (2024) reported a positive impact of personalized guidance on adherence only, with a higher completion rate of the IBI observed for patients receiving personalized human guidance. Sundström et al. (2016)'s results cannot be properly interpreted, because the choice and standard guidance groups were not compared directly against each other, instead they were combined and tested against self-help without any guidance. However, descriptively, both guidance groups showed comparable means in outcomes and adherence rates.

**Table 4:** Summary table of personalization of guidance.

Study	Target Population	Personalization Mechanism	Basis	Comparison Condition	Effect on Outcomes	Narrative Summary of Outcomes	Adherence Definition	Effect on Adherence	Narrative Summary of Adherence
Berger et al. (2011)	Social anxiety	Patient-requested contact after each lesson	Patient preference	Fixed weekly therapist contact	No significant effect	No difference in outcomes at post-treatment with effect sizes close to zero	Different engagement metrics: number of modules completed; time spent; exercises completed; use of collaborative elements	No significant effect	No differences between guided (standard) and step-up (personalized) conditions in lessons completed (M = 4.5 vs. 4.6), time spent (M = 10 h vs. 10h30m), exercises completed, or use of collaborative features.
Kleiboer et al. (2015)	Depression	Patient-requested contact after lessons	Patient preference	Standard weekly contact	No significant effect	No significant differences between standard frequency of weekly support and personalized (support on demand) guidance frequency	Number of modules completed	No significant effect	No difference in adherence between groups: 33 % (standard) vs. 31 % (personalized) completed all lessons; both groups completed 2.9 lessons on average.
Koelen et al. (2024)	anxiety and/or depression	Personalized human feedback adapted to user input	User Input	Standard feedback	No significant effect	No significant interaction of time x group when comparing personalized to standard guidance group in depression or anxiety	Number of modules completed, % of treatment completers	Higher adherence in personalized condition	Personalized feedback group showed significantly higher adherence than non-personalized group: more sessions completed (M = 3.32 vs. 2.54) and higher completion rates (26.9 % vs. 15.5 %).
Nissling et al. (2021)	anxiety disorders	Patient-selected IBI, modules, order, guidance modality & frequency	Patient preference	standard IBI program for anxiety disorders with standard weekly written guidance	Significant difference in anxiety	Significant difference in anxiety at post-treatment but not on other symptoms. At a 3-month follow up personalized group showed larger reduction of symptoms in the observed values, with between-group effects of d = 0.33 for anxiety symptoms, d = 0.47 for depression and d = 0.56 for general disability.	% of modules completed	No significant effect	% of modules completed appeared comparable between groups: 12 participants (48 %) in the personalized group completed 75 % to 100 % of modules compared with 14 (50 %) in the standard group.
Sundström et al. (2016)	Alcohol use disorder	Choice of guidance modality (chat vs. messaging)	Patient preference	Asynchronous text	Not interpretable (no direct comparison)	No direct comparison between personalized (choice) and standard guidance; both showed reduced AUDIT scores (personalized: M = 15.1, SD = 5.7; standard: M = 13.8, SD = 4.7) and alcohol consumption (personalized: M = 11.6 glasses, SD = 13.8; standard: M = 10.0 glasses, SD = 9.7).	Number of module exercises (number of written and saved exercise entries)	Not interpretable (no direct comparison)	Personalized choice group completed descriptively slightly more exercises (M = 4.4, SD = 2.5) than standard (messages) group (M = 3.7, SD = 2.7)
Zagorscak et al. (2018)	Depression	Personalized human feedback adapted to user input	User Input	Automated feedback	No significant effect	No significant between-group difference in primary or secondary outcomes	% of treatment completers (started seventh module)	Higher adherence in personalized condition	The personalized feedback group showed significantly higher completion rates (82.7 %) compared to the standard automated feedback group (74.2 %).

### 3.6 Personalization of communication

We did not identify any study investigating personalized communication vs. non-personalized communication, like personalized frequency of reminders vs. standard frequency of reminders.

### 3.7 UX

We identified two studies investigating a personalized UX. Table 5 provides a summary table of the personalization characteristics and overview of findings. Six et al. (2022) compared the customization of an avatar, its name, and the color of a hot-air balloon that was used regularly as a vehicle to “travel” between treatment modules to standard versions in a gamified IBI for depressed college students. Kelders et al. (2015) conducted a factorial study and varied several aspects in an IBI for depression. While their main goal was to compare fully individualized to automated guidance, they also investigated personalization of success stories: Participants in the high tailored arms were shown a success story of a person of the same gender and age group, who has the same symptoms as they have and the same reason for participating in the web-based intervention. Neither Six et al. (2022) nor Kelders et al. (2015) reported any positive impacts of the personalization on outcome or adherence.

**Table 5:** Summary table of personalization of UX.

Study	Target Population	Personalization Mechanism	Basis	Comparison	Effect on Outcomes	Narrative Summary of Outcomes	Adherence Definition	Effect on Adherence	Narrative Summary of Adherence
Kelders et al. (2015)	Depression	Personalized success stories matched on age, gender, symptoms	User profile	Standard success stories	No significant difference	No differences on clinical outcomes (note: high. vs. non- tailored not reported separately)	Completion of 9 lessons via log files	No significant difference	No differences on adherence (but high. vs. non-tailored not reported separately)
Six et al. (2022)	Depression	Customization of avatar, name, and interface visuals	User input	Standard avatar and visual design	No significant difference	The customized app did not lead to reduced depression or secondary outcomes in comparison to the standard app.	Different engagement metrics: Logins, journal entries, module completion	No significant difference	Descriptively more modules completed in customization group (M = 6.92 vs. 6.59; p = .07), with similar login activity (M = 0.51 vs. 0.48; p = .95) and journal entries (M = 6.13 vs. 6.41; p = .61).

## 4 Discussion

This systematic review aimed to provide a comprehensive overview of how personalization of treatment components impacts outcomes and adherence in IBIs for mental disorders. We identified thirteen studies comparing personalized to non-personalized components, with most studies investigating personalization of content and guidance. Personalization is a buzzword in the field, but the few existing studies do not yet provide clear evidence of the additive effect that personalization may have on outcomes or adherence. Hornstein et al. (2023)'s review highlighted that personalization is very frequently applied in IBIs for depression, from providing individualized guidance to receiving a personalized frequency of reminders when not logging into the intervention. However, our systematic review revealed that the additive effect of personalization over standard components is not well-studied.

Looking at the different components in isolation, the evidence regarding personalization's additional effects appears weak. The only study that reported a positive effect of personalization on anxiety outcomes is Nissling et al. (2021). However, in this study patients could not only tailor one component but several components to their needs. When personalization is limited to isolated components, the resulting effects may be too small to detect without large-scale trials, which poses practical challenges for research design. At the same time, in Nissling et al. (2021)'s study, it is unclear whether it is the extent of personalization (more is more) or whether it is the autonomy-fostering stance of the studied IBI that allowed patients to actively engage in selecting and adapting treatment content that has contributed to this finding. On closer inspection, patients in the patient-driven tailored treatment did not choose components that differed substantially from standard care. This raises the questions of mechanisms underlying positive personalization effects, like patients' expectation of personalized treatments working better or increased self-efficacy by patients taking their treatment into their own hands. Expectations and self-efficacy are discussed as important mechanisms of change in IBIs but their role in regard to personalization has not yet been investigated (Behr et al., 2025; Pontén et al., 2023). For psychological interventions more broadly, one meta-analysis has revealed small effects in favor of personalized interventions (Nye et al., 2023). None of the studies included in our review were adequately powered to detect effects of this size, with typical sample sizes insufficient to capture subtle but potentially meaningful personalization effects. This limitation underscores the need for larger, well-powered trials to better understand the true impact of different personalization strategies.

Personalized guidance entailed personalized timing, modality, and/or content of the feedback. None of the included studies reported a positive impact of personalized guidance on outcome. We included two studies that compared different support frequencies, standard weekly support and support provided on demand (Berger et al., 2011; Kleiboer et al., 2015). We chose to include these studies in our review because patients in the support-on-demand group were asked after every lesson/module whether they needed support. According to the Estimand Framework outlined in the ICH E9 (R1) guideline, contact-on-demand in these studies could be conceptualized as part of the treatment regimen (Heinrich et al., 2024). In other studies, however, contact-on-demand could be considered an intercurrent event, as it is not explicitly encouraged. These studies illustrate that, depending on how the contact-on-demand option is presented and communicated to patients, it could be viewed as a personalization of the frequency of support. Viewing contact-on-demand as a patient-driven, flexible form of guidance highlights its potential to address individual patient needs. This framing may help explain why studies comparing scheduled guidance with contact-on-demand often report comparable effectiveness (Koelen et al., 2022).

Zagorscak et al. (2018) and Koelen et al. (2024) demonstrated that individually tailored feedback messages can improve adherence. This is in line with guidance leading to better adherence than unguided treatments (Baumeister et al., 2014; Musiat et al., 2022). Musiat et al. (2022) defines guidance's aim “to support the clinical aspects of the intervention, facilitate intervention completion and/or achieve the desired clinical outcomes” (p. 230). This definition of guidance highlights that guidance varies greatly between studies - from highly individualized feedback to mainly adherence-fostering messages. Personalizing the content of guidance may hold potential for fostering a stronger therapeutic alliance between patients and online therapists. While positive associations between the alliance and outcome have been found in IBIs (Kaiser et al., 2021), it is an overall neglected area of IBI research (Zilcha-Mano and Fisher, 2022). Borrowing from concepts in conventional psychotherapy, Debrot et al. (2022) recently proposed a motive-oriented approach to guidance, offering a potential framework for tailoring guidance in IBIs. Future research should explore whether highly personalized guidance can indeed enhance therapeutic alliance, improve adherence, and ultimately lead to better outcomes.

In line with Matthews and Rhodes-Maquaire (2024) scoping review on personalization in mental health apps, the included studies in our reviews also implemented personalization of IBI components rather static. Personalization was typically determined at the outset of treatment without accounting for evolving needs or dynamic changes throughout the therapeutic process. Introducing a more dynamic approach to personalization — where adjustments are made in real-time based on patient feedback, progress, and changing circumstances — could allow for more adaptive and responsive interventions, leveraging the capabilities technology could offer (Wang and Miller, 2020). Incorporating passive sensing and artificial intelligence could further enhance this dynamic personalization by continuously monitoring behavioral, physiological, or contextual data to identify subtle changes in the patient's state. These insights could enable real-time tailoring of interventions, ensuring they remain responsive and aligned with the individual's evolving needs. While the use of AI in mental health interventions is rapidly expanding, implementing real-time AI-based personalization still remains challenging due to ethical, technical, and resource-related constraints. In particular, real-time personalization requires secure data infrastructure, continuous monitoring capabilities, and advanced algorithms, all of which may exceed the current resources available to most researchers conducting studies on IBIs.

Hornstein et al. (2023) have argued that customization, such as adjustments to visual features, does not fundamentally alter the therapeutic ingredients of an intervention and, therefore, may not constitute true personalization. However, the personalization of UX aspects may still represent a relevant dimension that warrants attention, as they may support the active ingredients and foster therapeutic engagement. While we have identified two studies investigating personalization in this dimension, neither reported a positive impact of personalization on outcome or adherence. Notably, Six et al. (2022) found that stronger identification with and perceived connection to an avatar were significantly associated with greater symptom reduction, though limited customization options may have constrained these effects. These findings point to a broader issue: Intervention design remains a relatively neglected area in personalization research (Idrees et al., 2024). Design features that enhance user identification and fit their context and preferences, whether through visual elements, interaction style, or narrative tone, may be critical to how users engage with digital interventions. Integrating personalization efforts with principles of good design, for instance by co-designing interventions with users to ensure a better match between users and IBIs (Orlowski et al., 2016; Schouten et al., 2022) as well as applying persuasive design principles (Idrees et al., 2024), may be key to increase adherence. In the context of VR, studies have explored the use of highly personalized avatars for diagnostic purposes or therapeutic activities, such as virtual mirror exposure (Di Natale et al., 2024). This emerging area of research suggests a need to further investigate how personalization could enhance engagement and outcomes.

Overall, all included studies were rated as having at least some concerns regarding their risk of bias. Several common sources of bias identified in the included studies reflect broader challenges in the e-mental health field, such as limited use of blinding, reliance on self-report measures, and high rates of missing data. Additionally, the review highlights an opportunity for growth in the adoption of open science practices, as none of the included studies preregistered or publicly shared a statistical analysis plan prior to conducting analyses. During the screening and data extraction process, we also observed that descriptions of personalization in the interventions were generally lacking in detail, making it challenging to classify personalization across different levels or to understand how opportunities for personalization were communicated to patients. Moreover, some studies treated personalization as an incidental by-product rather than a primary focus. Harnas et al. (2024) have suggested guiding questions on how personalization of interventions should be described in studies. Extending their framework to the specific context of IBIs is recommended.

Our review's scope was deliberately focused as we wanted to compare personalized to standard components. We, thus, excluded studies that compared personalized IBI components to other control groups (e.g., Carlbring et al., 2011; Dahlin et al., 2022; Silfvernagel et al., 2018) or that compared different mechanisms of personalization (Andersson et al., 2023). For instance, a recent study compared clinician vs. patient choice of IBI modules (Andersson et al., 2023). This highlights not only the importance of determining whether personalization is beneficial but also understanding which mechanisms of personalization are most effective. Further trials exploring these mechanisms are warranted to advance the field. Other excluded studies investigated personalization among a range of adherence-fostering measures, which would not allow researchers to draw conclusions on the effects of personalization (Jelinek et al., 2023). Future reviews may take a broader approach by mapping the full range of personalization strategies across IBIs, including those not directly compared to standard components, to provide a more comprehensive understanding of current practices. Such reviews could also synthesize evidence on mechanisms of action, implementation feasibility, and user acceptability.

As evident from the findings of our systematic review, personalization research in IBIs is still in its early stages. While there are clear limitations - such as the tension between user preferences and evidence-based practices, or the impracticality of tailoring interventions across an unlimited number of features - this should not lead to a pessimistic view of personalization's potential. IBIs offer a promising setting for personalization, given their modular structure, reusability of content, and capacity to integrate diverse data sources. However, this potential remains underrealized, as intervention content and design resources are often inaccessible to other research groups. Advancing personalization will therefore also depend on increased openness in intervention development, infrastructure, and collaboration across the e-mental health field. Designing scalable and effective IBIs requires strategic decisions about which elements to personalize and to what extent, rather than aiming for limitless individualization. Thus, it is important to investigate not just whether personalization is effective, but how and under what circumstances. Personalization may not enhance outcomes for all patients. First evidence suggests that tailoring content may be particularly beneficial for more complex cases, such as individuals with greater symptom severity or comorbid conditions (Johansson et al., 2012). Moreover, it is also crucial to explore the impact of personalization on mental health outcomes and adherence not only in the short-term but also in the long-term as well as explore impacts on broader markers such as patient satisfaction, quality of life, and mechanisms like therapeutic alliance.

This review was preregistered with the aim of exploring the impact of personalization in a nuanced way, including what was personalized, at what level, and through which mechanisms. Given the considerable heterogeneity across studies, a meta-analysis was not appropriate at this stage, as mixing these dissimilar studies probably creates “apples with oranges”-comparisons with questionable validity (Sharpe, 1997). Meta-analytic summaries will be more valuable as the evidence base grows and should be a goal for future research. Our systematic review revealed a gap between the theoretical potential of personalization in IBIs and the current evidence supporting its impact on outcomes and adherence. The diversity in personalization strategies across studies further complicates the ability to draw definitive conclusions. To make studies more comparable with each other, more detailed descriptions of how personalization is both implemented and communicated to patients are recommended.

## Declaration of Generative AI and AI-assisted technologies in the writing process

During the preparation of this work the authors used ChatGPT to improve clarity and readability for some individual sentences (OpenAI, 2024). After using this tool, the authors reviewed and edited the content as needed and take full responsibility for the content of the published article.

## Funding sources

This work was supported by the 10.13039/501100001659Deutsche Forschungsgemeinschaft (DFG, German Research Foundation)-Project-ID 422744262-TRR 289. Open Access Funding provided by Freie Universität Berlin.

## Declaration of competing interest

The authors declare that they have no known competing financial interests or personal relationships that could have appeared to influence the work reported in this paper.

## Appendix A Supplementary data

Supplementary material Image 1
