# Supplementary material for: A systematic review on personalization of treatment components in IBIs for mental disorders
Source: Internet Interv. 2025 Jun 14;41:100840. doi: 10.1016/j.invent.2025.100840 (PMC12205812; doi:10.1016/j.invent.2025.100840)
Supplement: Supplementary file 1 — Supplementary material [file mmc1.docx]

Appendix for

“Systematic Review on the Effects of Personalization of Treatment Components in Internet-based Interventions for Mental Disorders”

Search Strings

Table S1. Search Strings for all Databases that were included in the Systematic Search

|  | **Search String** |
| --- | --- |
| **Pubmed** | ("transdiagnostic"[tw] OR "personali*"[tw] OR "tailor*"[tw] OR "individuali*"[tw] OR "choice"[tw] OR "customi*"[tw]) AND ("ICBT"[tw] OR "internet-based"[tw] OR "internet"[tw] OR "web-based"[tw] OR "web"[tw] OR "digital"[tw] OR "digital technology"[MeSH Terms] OR "mobile applications"[MeSH Terms] OR "Smartphone"[MeSH Terms]) AND ("randomized controlled trial"[tw] OR "randomised controlled trial"[tw] OR "RCT"[tw] OR "control group"[tw] OR "control condition"[tw]) AND ("anxi*"[tw] OR "depress*"[tw] OR "emotional disorder*"[tw] OR "mental health"[tw] OR "mental disorder"[tw]) |
| **PsycINFO & Medline** | TX("transdiagnostic" OR "personali*" OR "tailor*" OR "individuali*" OR "choice" OR "customi*") AND TX("ICBT" OR "internet-based" OR "internet" OR "web-based" OR "web" OR "digital" OR "digital technology" OR "mobile applications" OR "smartphone") AND TX("randomized controlled trial" OR "randomised controlled trial" OR "RCT" OR "control group" OR "control condition") AND TX("anxi*" OR "depress*" OR "emotional disorder*" OR "mental health" OR "mental disorder") |
| **Google Scholar** | (intext:"transdiagnostic" OR intext:"personali*" OR intext:"tailor*" OR intext:"individuali*" OR intext:"choice" OR intext:"customi*") AND (intext:"ICBT" OR intext:"internet-based" OR intext:"internet" OR intext:"web-based" OR intext:"web" OR intext:"digital") AND (intext:"randomized controlled trial" OR intext:"randomised controlled trial" OR intext:"RCT" OR intext:"control group" OR intext:"control condition") AND (intext:"anxi*" OR intext:"depress*" OR intext:"emotional disorder*" OR intext:"mental health" OR intext:"mental disorder") |

**Table S2**

*Reasons For Exclusion for Individual Studies*

|  | **Reason for Exclusion** | | | | | | | | |  |  |
| --- | --- | --- | --- | --- | --- | --- | --- | --- | --- | --- | --- |
|  | other language | no RCT | no adults | no standalone IBI | no elevated mental health symptoms | primary focus on somatic disorders | no personalized component | wrong control group | wrong outcome | other | comments |
|  |  |  |  |  |  |  |  |  |  |  |  |
| Aardoom et al. (2016) |  |  | X |  |  |  |  | X |  |  | individuals aged 16-18 |
| Aguilera et al. (2024) |  |  |  |  |  |  |  |  | X |  |  |
| Andersson et al. (2023) |  |  |  |  |  |  |  | X |  |  | comparison between self-tailored and clinician-tailored |
| Andrews et al. (2023) |  |  |  | X |  |  |  | X |  |  | video chat support; High or low therapist support |
| Anmella et al. (2024) |  | X |  |  |  |  |  | X |  |  | no RCT published yet; phase 2 trial is testing the effects of PRESTOapp, however there is no standardized control group |
| Augsburger et al. (2022) |  |  |  |  |  |  |  | X |  |  |  |
| Batterham et al. (2021) |  |  |  |  |  |  |  | X |  |  | no standard equivalent |
| Benjet et al. (2023a) |  |  |  |  |  |  |  | X |  |  |  |
| Benjet et al. (2023b) |  |  |  |  |  |  | X | X |  |  |  |
| Bennett (2022) |  |  |  |  | X |  | X | X |  |  |  |
| Bergman Nordgren (2013) |  |  |  |  |  |  |  | X |  |  |  |
| Bertholet et al. (2018) |  |  |  |  |  |  |  | X |  |  |  |
| Beyer et al. (2014) |  |  |  |  |  |  |  | X |  |  |  |
| Boettcher et al. (2014) |  |  |  |  |  |  | X | X |  |  |  |
| Boon et al. (2011) |  |  |  |  |  |  |  | X |  |  | CG: only read standard brochure |
| Boß et al. (2018) |  |  |  |  |  |  |  | X |  |  | both intervention groups got personalized normative feedback |
| Braun et al. (2021a) |  |  |  |  |  |  |  | X |  |  |  |
| Braun et al. (2021b) |  |  |  |  |  |  |  | X |  |  |  |
| Buntrock et al. (2022) |  |  |  |  |  |  |  | X |  |  |  |
| Campos et al. (2019) |  |  |  |  |  |  |  | X |  |  |  |
| ‍Carlbring et al. (2011) |  |  |  |  |  |  |  | X |  |  |  |
| Clarke et al. (2014) |  |  |  |  |  |  |  | X |  |  | secondary analysis myCompass (CG: attention control) |
| Cook et al. (2019) |  |  |  |  | X |  |  | X |  |  |  |
|  |  |  |  |  |  |  |  |  |  |  |  |
| Cunningham et al. (2010) |  |  |  |  |  |  |  | X |  |  |  |
| Cunningham et al. (2012) |  |  |  |  |  |  |  | X |  |  | in CG: only all normative comparison information was removed |
|  |  |  |  |  |  |  |  |  |  |  |  |
| Cunningham et al. (2014) |  |  |  |  |  |  |  | X |  |  |  |
| Cunningham et al. (2021a) |  |  |  |  |  |  | X | X |  |  | group-level tailoring |
| Cunningham et al. (2021b) |  |  |  |  |  |  | X |  |  |  |  |
| Dear et al. (2015) |  |  |  |  |  |  |  | X |  |  | contact on demand was not encouraged weekly |
| Dear et al. (2016) |  |  |  |  |  |  |  | X |  |  | contact on demand was not encouraged weekly |
| Dear et al. (2018) |  |  |  |  |  |  | X | X |  |  |  |
| Dietel et al. (2020) |  |  |  |  |  |  | X | X |  |  | with and without feedback |
| Ditton et al. (2023) |  |  |  |  | X |  |  |  |  |  |  |
| Eberle et al. (2023) |  |  |  |  | X |  | X | X |  |  |  |
| Elfeddali et al. (2012) |  |  |  |  |  |  |  | X |  |  |  |
| Fatouros et al. (2025) |  |  |  |  |  |  |  | X |  |  |  |
| Fitzsimmons-Craft et al. (2024) |  |  |  |  |  |  |  | X | X |  |  |
| Flygare et al. (2020) |  |  |  |  |  |  | X |  |  |  | no personalized component |
| Ford-Gilboe et al. (2020) |  |  |  |  | X |  |  |  |  |  |  |
| Frank et al. (2022) |  |  |  | X |  |  |  | X |  |  |  |
| Gajecki (2022) |  |  |  |  |  |  |  |  |  |  | same as: Guided and Unguided Internet-Based Treatment for Problematic Alcohol Use – A Randomized Controlled Pilot Trial |
| Gander et al. (2013) |  | X |  |  | X |  | X | X |  |  |  |
| Garey et al. (2021) |  |  |  |  |  |  | X | X |  |  | group-level tailoring |
| González-Robles et al. (2020) |  |  |  |  |  |  | X | X |  |  |  |
| Hadjistavropoulos et al. (2017) |  |  |  |  |  |  |  | X |  |  | Contact on demand not encouraged weekly |
| Hodgins et al. (2019) |  |  |  |  |  |  |  | X |  |  | not the same intervention |
| Hudson et al. (2017) |  |  |  |  |  |  |  | X |  |  |  |
| Jagayat et al. (2024) |  |  |  |  |  |  |  | X |  |  | control group received same IBI but without dynamic tailoring; personalization not isolated. |
| Jelinek et al. (2023) |  |  |  |  |  |  |  | X |  |  | personalization was part of a range of adherence-fostering measures that the control group did not receive |
| Jin (2018) |  |  |  |  |  |  | X | X |  |  | user choice of modules in both groups; CG: only cultural adaptation of myCompass |
| Jo et al. (2019) |  |  |  |  |  |  |  | X |  |  |  |
| Jung et al. (2024) |  |  |  | X |  |  |  |  | X |  | anxiety levels after VR exposure (higher in personal group), but not about effectiveness of treatment |
| Karyotaki et al. (2022) |  |  |  |  |  |  |  | X |  |  |  |
| Kladnitski et al. (2020) |  |  |  |  |  |  | X | X |  |  |  |
| Kohlmann et al. (2024) |  |  |  | X |  |  |  | X |  |  |  |
| Kordy et al. (2013) |  |  |  | X |  |  |  |  |  |  |  |
| Krieger et al. (2019) |  |  |  |  |  |  | X | X |  |  |  |
| LaBrie et al. (2013) |  |  |  |  |  |  | X | X |  |  | group-level tailoring |
| Lai et al. (2025) |  | X |  |  |  |  |  |  |  |  | Study protocol only |
| Lancee et al. (2013) |  |  |  |  |  |  |  | X |  |  |  |
| Leightley et al. (2022) |  |  |  |  |  |  |  | X |  |  |  |
| López-del-Hoyo et al. (2013) |  |  |  |  |  |  |  | X |  |  |  |
| Luther et al. (2020) |  |  |  |  |  |  |  | X |  |  |  |
| Martínez-Vispo et al. (2024) |  | X |  |  |  |  |  |  |  |  |  |
| Mira et al. (2017) |  |  |  |  |  |  | X | X |  |  | no standardized equivalent for human support |
| Mistretta et al. (2018) |  |  |  | X |  |  | X | X |  |  |  |
| Mohr et al. (2013) |  |  |  |  |  |  |  | X |  |  | no standardized equivalent for TeleCoaching |
| Montero-Marín et al. (2016) |  |  |  |  |  |  |  | X |  |  |  |
| Moore et al. (2018) |  |  |  |  | X |  | X |  | X |  | ART Reminder and Reinforcement Text Messages only repeated participants' input; METH use messages were not personalized at all; focus on medication adherence; control group did not receive any text messages |
| Moritz et al. (2016) |  |  |  | X |  |  |  |  |  |  | the study is conducted via the internet with self-help PDFs - not an IBI per se |
| Muench et al. (2023) |  |  |  |  |  |  |  | X |  |  | both intervention groups were tailored, no equivalent for interactive text messages in TA group |
| Muth et al. (2021) |  |  |  |  |  |  | X | X | X |  |  |
| Neighbors et al. (2010) |  |  |  |  |  |  |  | X |  |  | intervention groups differed in personalization regarding their gender -> no individual personalization |
| Nomeikaite et al. (2025) |  |  |  |  | X |  |  | X |  |  |  |
| Nordgren et al. (2014) |  |  |  |  |  |  |  | X |  |  | attention control (clients were asked about their well-being weekly) |
| ‍Nordgren et al. (2024) |  |  |  |  |  |  |  | X |  |  |  |
| Pachankis et al. (2020) |  |  |  |  | X |  | X | X |  |  | "presented vignettes were identical across participants, although they were matched to each participant’s sexual identity, gender identity, and race/ethnicity" |
| Papinczak et al. (2021) |  |  |  | X | X |  |  | X | X |  |  |
| Patrick et al. (2023) |  |  |  |  | X |  |  | X |  |  | preventive intervention |
| Probst et al. (2020) |  |  |  |  |  |  |  | X |  |  |  |
| Rabius et al. (2008) |  |  |  |  |  |  |  | X |  |  |  |
| Rocha (2013) |  |  |  |  |  |  |  | X |  |  |  |
| Romero-Sanchiz et al. (2017) |  |  |  |  |  |  |  | X |  |  | no standardized equivalent for personalized guidance |
| Ruiz-Yu et al. (2024) |  | X |  | X |  |  |  |  |  |  |  |
| Schaub et al. (2019) |  |  |  |  |  |  |  | X |  |  |  |
| Schlosser et al. (2018) |  |  | X |  |  |  |  | X |  |  |  |
| Schulz et al. (2013) |  |  |  |  |  |  |  | X |  |  |  |
| Schulz et al. (2014) |  |  |  |  | X |  |  | X | X |  |  |
| Sharma et al. (2022) |  |  |  |  |  |  |  | X |  |  |  |
| Shelton (2019) |  |  |  | X |  |  |  |  |  |  | they investigated a proposed IBI, it is not an actual treatment study |
| Shen et al. (2025) |  |  |  |  | X |  |  |  |  |  | no current drug use as inclusion criterion |
| Silfvernagel et al. (2018) |  |  |  |  |  |  |  | X |  |  |  |
| Smit et al. (2013) |  |  |  |  |  |  |  | X |  |  |  |
| Smit et al. (2016) |  |  |  | X |  |  |  | X |  |  |  |
| Stanczyk et al. (2014a) |  |  |  |  |  |  |  | X |  |  | Computer Tailored (CT) smoking cessation interventions (video- vs text-based) compared to CG (general text-based advice) |
| Stanczyk et al. (2014b) |  |  |  |  |  |  |  | X |  |  | tailoring group with video messages, a text computer tailoring group with text messages, CG with short generic text advice |
| Stentzel et al. (2021) |  |  |  |  |  |  |  | X | X |  |  |
| Suchan et al. (2022) |  |  |  |  |  |  |  | X |  |  |  |
| Suffoletto et al. (2021) |  |  |  |  |  |  |  | X |  |  |  |
| Sun et al. (2022) |  |  |  |  |  |  | X | X |  |  | not personalized; mindfulness-based mHealth compared to time- & attention-matched social support-based mHealth CG |
| Sundström et al. (2016) |  |  |  |  |  |  |  | X |  |  | all groups received automated feedback after completing a module, only the guided intervention group received feedback from therapist |
| Sundström et al. (2020) |  |  |  |  |  |  | X |  |  |  | allocated to AHC with brief health educator guidance vs Allocated to AHC without brief health educator guidance |
| Takeuchi et al. (2024) |  | X |  |  |  | X |  | X |  |  |  |
| Thorisdottir et al. (2021) |  |  |  |  | X |  | X | X | X |  | targets the negative impact of bullying |
| Titov et al. (2015) |  |  |  |  |  |  | X | X |  |  | internet delivered TD-CBT / DS-CBT intervention delivered in either clinician-guided (CG-CBT) or self-guided (SG-CBT) formats |
| Titov et al. (2016) |  |  |  |  |  |  |  | X |  |  |  |
| Tregarthen et al. (2019) |  |  |  |  | X |  |  | X |  |  | no standardized equivalent for personalized components in Standard App Intervention |
| van Beugen et al. (2016) |  |  |  |  |  | X |  | X |  |  |  |
| van Lettow et al. (2015) |  |  |  |  |  |  |  | X |  |  | no standardized equivalent for feedback in prototype and combined conditions; all conditions received feedback tailored to "demographic background [...], alcohol consumption and intentions to reduce drinking" |
| Vandelanotte et al. (2022) |  |  |  |  | X |  |  | X |  |  |  |
| Vernmark et al. (2010) |  |  |  |  |  |  |  | X |  |  | patients in the email therapy group did not receive self-help content |
| Wang and Farb (2023) |  |  |  |  | X |  |  |  | X |  |  |
| Weiner et al. (2024) |  |  |  |  |  |  |  | X |  |  |  |
| Wen et al. (2024) |  |  |  |  |  |  | X |  |  |  |  |
| White and Pohl (2022) |  |  |  |  |  |  | X | X |  |  |  |
| White et al. (2020) |  |  |  | X | X |  |  |  |  |  |  |
| Zarski et al. (2024) |  |  |  |  |  |  |  |  |  | X | only subclinical symptoms; individuals with clinical diagnoses were excluded |
| Zagorscak et al. (2020) |  |  |  |  |  |  |  |  | X |  | secondary analysis |
